# Intravenous lipid emulsion in clinical toxicology

**DOI:** 10.1186/1757-7241-18-51

**Published:** 2010-10-05

**Authors:** Leelach Rothschild, Sarah Bern, Sarah Oswald, Guy Weinberg

**Affiliations:** 1 Department of Anesthesiology, University of Illinois at Chicago, UIC Medical Center, Chicago, Illinois, USA; 2 Jesse Brown VA Medical Center, Chicago, Illinois, USA

## Abstract

Intravenous lipid emulsion is an established, effective treatment for local anesthetic-induced cardiovascular collapse. The predominant theory for its mechanism of action is that by creating an expanded, intravascular lipid phase, equilibria are established that drive the offending drug from target tissues into the newly formed 'lipid sink'. Based on this hypothesis, lipid emulsion has been considered a candidate for generic reversal of toxicity caused by overdose of any lipophilic drug. Recent case reports of successful resuscitation suggest the efficacy of lipid emulsion infusion for treating non-local anesthetic overdoses across a wide spectrum of drugs: beta blockers, calcium channel blockers, parasiticides, herbicides and several varieties of psychotropic agents. Lipid emulsion therapy is gaining acceptance in emergency rooms and other critical care settings as a possible treatment for lipophilic drug toxicity. While protocols exist for administration of lipid emulsion in the setting of local anesthetic toxicity, no optimal regimen has been established for treatment of acute non-local anesthetic poisonings. Future studies will shape the evolving recommendations for lipid emulsion in the setting of non-local anesthetic drug overdose.

## Introduction

Intravenous lipid emulsion (ILE) is a novel method for treating local anesthetic systemic toxicity (LAST) that also shows promise as an effective antidote for other lipophilic drug poisonings. Cardiovascular collapse is the most life-endangering complication of local anesthetic (LA) absorption or intravascular injection during regional anesthesia [1]. LAST is generally considered to be resistant to conventional modes of resuscitation. However, in 1998, Weinberg et al. reported the effective use of a lipid emulsion infusion in resuscitation of bupivacaine overdose in rats [2]. Follow up studies in dogs confirmed the efficacy of ILE in treating an otherwise fatal overdose of bupivacaine, even after an interval of 20 minutes [3]. Subsequent case reports demonstrated rapid reversal of LAST with use of ILE often after standard resuscitative efforts had failed [4,5]. Lipid therapy has also been utilized in patients suffering from poisonings other than those involving LA toxicities [6,7]. Recent research has focused on the efficacy of lipid emulsion in resuscitating patients from overdoses of lipophilic, non-LA agents. This article focuses on the history of lipid resucitation, its theorized mechanisms of action, and its use in local and non-LA drug overdoses. Relevant articles were gathered by the authors' independent searches of multiple bibliographic databases. Administration of any formulation of ILE and a range of outcome measures (mortality, hemodynamics, mental status, cardiac function, adverse effects) were considered.

## Last: Discovery and Evolution of ILE

### Development of ILE

Cardiotoxicity resulting from bupivacaine and other local anesthetics has been the subject of laboratory investigation for over three decades. Long acting lipophilic local anesthetics such as bupivacaine and etidocaine were implicated in several fatal cardiac arrests reported in 1979 by Albright [8]. These events all appeared to resist standard forms of resuscitation. ILE was initially identified as an antidote for LAST, the most feared complication of regional anesthesia. In 1997, Weinberg et al. described a patient with severe carnitine deficiency who suffered a cardiac arrest from only 22 mg of bupivacaine administered subcutaneously with injection of tumescent solution during a general anesthetic [9]. This case led to studies of the potential interaction of bupivacaine and elements of the carnitine cycle that later confirmed bupivacaine potently inhibits the mitochondrial enzyme carnitine-acylcarnitine translocase [10]. This observation led to animal studies that ultimately identified the benefit of ILE resuscitation.

**Figure 1:**
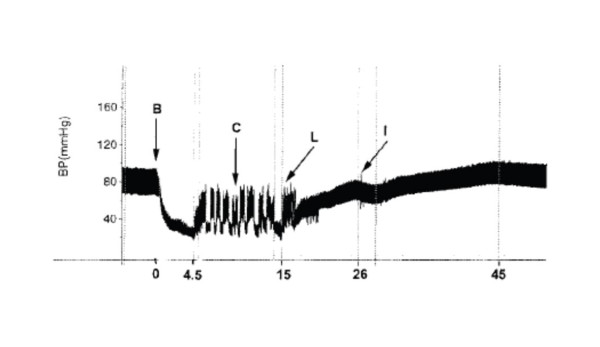
**BP during a typical experiment**. B indicates the start of a bupivacaine 10 mg/kg bolus. This is taken as zero time. Criteria for circulatory collapse were reached at 4.5 minutes, and internal cardiac massage (indicated by C) was begun, causing the subsequent pressure spikes that continued until shortly after the lipid infusion (indicated by L), which began at 15 minutes. Circulation was sufficiently established by 26 minutes (after roughly 10 minutes of lipid therapy), when isoflurane general anesthesia was restarted (indicated by I).

Experiments demonstrated that rats pretreated with lipid became resistant to bupivacaine-induced cardiac effects, as a larger dose of bupivacaine was required to induce asystole [2]. Additional dose-response experiments in the same study found that when 20% lipid emulsion was given during resuscitation after the intravenous bupivacaine bolus dose (post-treatment) the LD50 of bupivacaine was increased from 12.5 mg/kg to 18 mg/kg. Moreover, the survival curves were sufficiently shifted at a bupivacaine dose of 15 mg/kg, where all control animals died while no deaths occurred in those animals given ILE. This observation suggested a potential for using ILE in treatment of cardiotoxicity resulting from local anesthetic toxicity. Follow-up experiments in the Weinberg lab examined the efficacy of ILE in anesthetized dogs after an intravenous overdose of bupivacaine (10 mg/kg) [3] [figure 1]. Resuscitation comprised open-chest cardiac massage with or without a 20% lipid infusion. All dogs receiving lipid infusion recovered normal blood pressure and EKG traces, while all control animals died.

### Optimization Studies and Remaining Questions

Investigations in the past several years have focused attention on the merits of ILE in the setting of standard resuscitation protocols. Weinberg et al studied lipid compared to epinephrine in a rat model of bupivacaine-induced cardiac arrest [11]. Ten minutes after initiating the resuscitation, the mean rate pressure product (RPP, systolic pressure × heart rate) in the lipid group was significantly greater than that in the epinephrine-treated group, which was no different than that of the saline controls. Moreover, the epinephrine-treated groups had worse metabolic indicators of recovery: the pH, PaO2, and SvO2 were all lower in the epinephrine group, and lactate levels were higher. Pulmonary edema occurred almost immediately in four of the five epinephrine-treated rats, but in none in the lipid group.

A follow-up study compared recovery following bupivacaine overdose in rats treated with ILE versus vasopressin either alone or combined with epinephrine [12]. ILE was more effective than either of the other treatments. Specifically, hemodynamic and metabolic parameters measured at 15 minutes, (five minutes after cessation of resuscitation) indicated a poor quality of recovery in all rats receiving vasopressin, with or without epinephrine. Metrics of tissue perfusion (e.g., central venous oxygen tension and blood lactate concentration) were particularly adversely affected by vasopressin. Moreover, lung wet-to-dry ratios were higher among the groups that received vasopressin compared with the ILE cohort, implying permeable increases in lung parenchyma and potential structural damage. A study of the dose-response to epinephrine during lipid treatment of bupivacaine overdose in rats indicated that while epinephrine routinely resulted in more rapid return of RPP than lipid (only) in the first few minutes of resuscitation, by the experiment's end, animals that had received 10 mcg/kg or more of epinephrine experienced substantial decline in all hemodynamic and metabolic parameters [13]. In addition, it was clear that high systolic pressures early in the resuscitation did not imply successful resuscitation by 15 minutes, at which time lipid-treated subjects exhibited better recovery profiles than those given the higher doses of epinephrine. These three studies beg the same question: "Is lipid emulsion superior to standard resuscitation protocols for cardiac arrest attributable to local anesthetic toxicity"? If so, does it follow that one should avoid or modify the recommendations for standard ACLS pressors in the setting of local anesthetic toxicity or other lipophilic drug overdoses, opting instead to use ILE alone as first-line treatment or resuscitation?

A study by Mayr et al[14] challenges the use of ILE compared with vasopressor treatment in bupivacaine overdose. In a porcine model, Mayr et al demonstrated that vasopressin plus epinephrine led to higher coronary perfusion pressure and better short-term survival as compared to lipid infusion [14]. However, in this model mechanical ventilation was discontinued after the injection of bupivacaine (5 mg/kg) and apnea was maintained until asystole occurred plus an additional minute. Therefore it is possible that the asphyxia was a confounding factor that diminished efficacy of the ILE and influenced the authors' interpretation of the data that vasopressor therapy is more effective than lipid. Similarly, in 2009 Hicks et al [15] demonstrated in a swine model, that lipid emulsion combined with epinephrine and vasopressin did not improve survival or hasten return of spontaneous circulation. However, these animals all received massive doses of epinephrine and vasopressin during a resuscitation interval of ten minutes prior to receiving the test treatments. Findings published from the Weinberg lab predict exactly this finding: that lipid emulsion may show little or no benefit when the subject has also received large vasopressor doses [13]. The cardiac anatomy and physiology of swine differs from canines and these species-specific differences might also contribute to the diminished effect of lipid emulsion in the setting of bupivicaine toxicity [16]. The optimal model for assessing treatment of bupivacaine toxicity will need to be identified for future studies to offer a more complete understanding of this phenomenon.

## ILE in Last

Many case reports [17-21] and animal studies [2,3,11,22] describe the successful use of ILE to reverse local anesthetic toxicity, which can present with neurologic symptoms with or without cardiovascular instability. One of the earliest cases describes the accidental injection of 40 ml of 1% ropivicaine for an axillary plexus block in an 84-year-old woman [20]. Shortly after the block was placed, the patient developed generalized tonic-clonic seizures followed by asystole. Standard resuscitation measures were unsuccessful and after 10 min of cardiovascular collapse, lipid emulsion was given by a bolus followed by an infusion. Normal EKG rhythm returned, and blood pressure was restored to normal; the patient was discharged to home in four days with near complete recovery. This sequence is typical of successful ILE: rapid reversal of toxicity after standard measures have failed. McCutchen et al [23] described the co-administration of standard ACLS drugs and ILE to rescuscitate an 82 year old woman who underwent sciatic block placement immediately after uneventful femoral nerve catheter placement and bolus. Within twenty seconds of bupivacaine injection for the sciatic block, the patient suffered a general clonic-tonic seizure which responded to midazolam. A patent airway was maintained, but the patient's rhythm converted to ventricular tachycardia and did not respond immediately to a single dose of amiodarone followed by a single lipid bolus. Ventricular tachycardia persisted for several minutes and one countershock was given followed by a continuous lipid infusion. Although initially obtunded, after two hours of infusion the patient's mental status returned to normal. The authors attribute the full recovery and the avoidance of cardiovascular collapse to rapid administration of lipid.

Cardiac toxicity is at times preceded by CNS symptoms and some physicians have chosen to administer ILE earlier in the progression of the toxicity syndrome. Many case reports describe the use of ILE prior to the onset of cardiovascular collapse [17,19,20,23]. For instance, Foxall et al[18] described the use of ILE to treat CNS toxicity and ventricular ectopy in an effort to prevent the progression to cardiac arrest. In another case, a 13 year old girl developed ventricular tachycardia after a lumbar plexus block with ropivicaine-lidocaine[24]. ILE was administered at the onset of this arrhythmia and normal vital signs were quickly restored. The EKG returned to baseline and surgery proceeded uneventfully. The evidence for efficacy of ILE's ability to reverse cardiac local anesthetic toxicity continues to mount.

## Mechanism of Action of ILE

### Lipid Sink Phenomenon

The solubility of long-acting local anesthetics in lipid emulsion and the high binding capacity of these emulsions likely explain the clinical efficacy when lipid is rapidly infused in cases of LAST. Initially coined in 1998 by Weinberg[2], the 'lipid sink' phenomenon is the most widely accepted mechanism of action for ILE. Lipid emulsion infusion creates an expanded lipid phase, and the resulting equilibrium drives toxic drug from tissue to the aqueous plasma phase then to the lipid phase.

**Figure 2:**
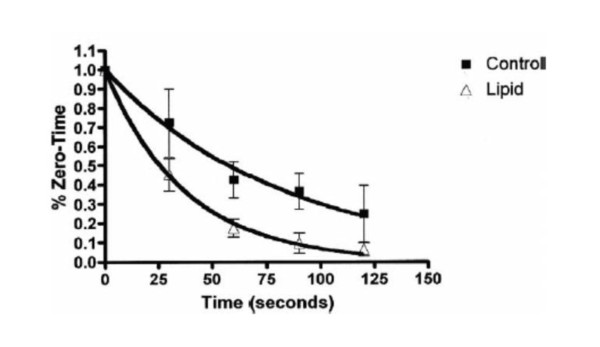
**Cardiac bupivacaine content**. The trends for myocardial bupivacaine content are shown during the 2 minutes after a 30-second infusion of bupivacaine 500 μmol/L for control and lipid-treated hearts. Values are normalized to zero time, and error bars indicate standard deviation (n = 5 for both groups). Regression curves were fitted by single exponential decay functions with time constants 83 seconds (R^2 ^= 0.9861) and 37 seconds (R^2 ^= 0.9978) for control and lipid groups, respectively.

While the exact mechanisms of action of lipid emulsion infusion to treat LAST remain unclear, the key component is likely the binding property of the emulsion[25]. The 20% Intralipid™(Kabivitrum Inc., California, USA) emulsion consists of 20% soybean oil, 1.2% egg yolk phospholipids, 2.25% glycerin, water and sodium hydroxide. Although Intralipid™is the most common commercial preparation used in documented resuscitations, there are many different ILE products with different formulations. It is nonetheless the emulsified fat droplets that form a lipid compartment, into which lipophilic substances are theoretically partitioned, when infused into an aqueous medium such as blood. Lipophilic substances, such as local anesthetics, are drawn into the "lipid sink" and a concentration gradient develops between tissue and blood which cause local anesthetics to move away from the heart or brain (areas of high concentrations) to the "lipid sink". In an experimental rat model, Weinberg et al demonstrated that radiolabeled bupivacaine added *in vitro *to lipid-treated rat plasma preferentially moves to the lipid phase with a partition coefficient of 11[2]. In subsequent experiments using an isolated heart model of bupivacaine toxicity, Weinberg et al showed that infusion with lipid emulsion accelerates the removal of radiolabeled bupivacaine from myocardial tissue compared with controls[26] [figure 2].

### Alternate Mechanisms

Lipid emulsion could theoretically increase intracellular fatty acid content and therefore overcome the reduced ATP production, which results from LA block of fatty acid transport and oxidation. It is possible that the resulting increased intracellular fatty acid content contributes to improved ATP synthesis in the cardiomyocyte. Under normal aerobic conditions, fatty acids are the preferred substrate for myocyte oxidative phosphorylation, generating about 80-90% of cardiac adenosine triphosphtae (ATP)[27]. If fatty acid transport is interrupted, then ATP production decreases, negatively impacting myocyte survival and potentially leading to cardiac toxicity. Van de Velde et al[28] used a dog model to demonstrate that infusion of 20% lipid emulsion improves contractility because of improved fatty acid oxidation. Another study performed by Eledjam et al[29] showed that pre-incubation with ATP in isolated myocardial strips prevents depression of contractility by bupivacaine. Therefore, ILE may increase intracellular fatty acid content enough to reverse or overcome the decrease in cardiac ATP synthesis.

Interestingly, lipid emulsion was initially observed as acting faster *in vivo *settings than was anticipated based on a simple lipid sink mechanism, implying that direct cardiotonic effects might also be at play[30]. Stehr et al[31] demonstrated that lipid emulsion reverses bupivicaine-induced contractile depression at concentrations that are too low to provide a lipid sink phenomenon, suggesting a metabolic explanation for the positive effect. Lipid emulsion infusion might also directly increase intramyocyte calcium levels and lead to a direct positive inotropic effect[32]. Fatty acids have also been shown to increase calcium levels in cardiac myocytes. Although the precise mechanisms of action of ILE treatment of LAST requires further elucidation, the key component is likely due to the efficient binding properties of the emulsion.

## ILE in Non-LA Drug Toxicity

Lipid emulsion therapy has not been limited to the treatment of local anesthetic toxicity. Because of recent human case reports of successful resuscitation, there has been increasing interest in the potential benefit of lipid emulsion in cardiac arrests attributable to lipophilic, non-LA drugs [33,34]. Two comprehensive literature reviews describe the use of ILE in the setting of non-LA overdoses [35,36]. Recent case reports of successful resuscitation suggest the efficacy of lipid emulsion infusion for treating non-local anesthetic overdoses across a wide spectrum of drugs: beta blockers, calcium channel blockers, parasiticides, herbicides and several varieties of psychotropic agents. The most clinically relevant of these are likely to be toxicities caused by tricyclic antidepressants and other psychotropic drugs, calcium channel blockers and beta blockers. These medications share similar sodium channel blocking properties with local anesthetics and are generally quite lipophilic. Presumably, ILE exerts the same "lipid sink" effect with these lipophilic drugs, thereby decreasing the amount of active drug in the target tissue and reducing toxicity[37].

### Psychotropic Drugs

Yoav et al [38] showed decreased mortality in rats when clomipramine was administered in a lipid infusion vehicle versus saline. Harvey and Cave [39] used a rabbit model to study the effects of lipid infusion for clomipramine toxicity and found faster recovery from hypotension in lipid-treated rabbits compared to saline or sodium bicarbonate treated controls. A rat model of amitriptyline toxicity failed to find statistically significant differences in hemodynamic parameters or survival, but these findings may be explained by the small sample size [40].

The first report of lipid emulsion's successful use in a human as an antidote for a lipophilic, non-local anesthetic toxicity was by Sirianni et al [6]. They describe the resuscitation of a 17 year old female after massive ingestion of bupropion and lamotrigine, prescribed for the treatment of depression and bipolar disorder. Ten hours later, the patient experienced complete cardiovascular collapse with ventricular fibrillation and pulseless electrical activity. After seventy minutes of unsuccessful resuscitation using standard ACLS plus sodium bicarbonate injection, 20% ILE was given as a last attempt to restore hemodynamic stability. Within one minute of ILE administration normal vital signs were re-established. She recovered and was discharged from the hospital with minimal neurologic deficits.

A case report in *Anaesthesia *described the use of ILE in a 61 year old male who intentionally ingested toxic levels of quetiapine and sertraline, a protein bound drug that is susceptive to ILE effects due to its high lipid partition co-efficient [41]. The patient presented to the emergency department with a Glasgow coma scale of 3 and in normal sinus rhythm with no QT prolongation, but hypotensive. Approximately four hours after ingestion, 20% lipid emulsion was given at a bolus dose of 1.5 mL/kg; within fifteen minutes, a rapid increase in the patient's level of consciousness was observed, to a GCS of 9, negating the need for intubation in this patient. All vitals were within normal ranges within 12 hours of admission, and the patient was subsequently discharged.

Weinberg et al [7] reported a case of successful resuscitation of a patient with haloperidol induced cardiac arrest. The patient was admitted to the hospital with an underlying prolonged QT interval on electrocardiogram and developed ventricular bigeminy with pulseless multiform ventricular tachycardia after haloperidol administration. After administration of lipid emulsion therapy, the patient's rhythm was restored; she was completely alert and oriented 18 hours after the event.

### Calcium Channel Blockers (CCBs)

Multiple animal trials have demonstrated the benefits of ILE versus placebo in verapamil toxicity [42]. Tebbutt et al [42] showed in 2006 that the use of lipid emulsion almost doubled the LD50 and attenuated the bradycardia seen with toxic doses of verapamil in rats. Bania et al [43] subsequently evaluated lipid emulsion compared to standard resuscitation techniques in a canine model of verapamil toxicity and found that ILE-treated animals had significantly higher MAPs at 30, 45, and 60 minutes post-rescue compared to control dogs. A confirmation of efficacy for ILE in treating CCB overdose was provided by Young et al [44] who published the first human case of verapamil toxicity successfully treated with lipid emulsion. Their patient was in shock that was refractory to standard resuscitation therapy but resolved with administration of intravenous lipid emulsion; no adverse events were noted and full patient recovery ensued. Other case reports relate similar hemodynamic improvement in patients with calcium channel blocker overdoses [45,46].

### Beta Blockers

In both rat and rabbit models, ILE mitigates propanolol-induced QRS prolongation and attenuates associated bradycardia [47,48]. A similar model exploring ILE in the treatment of atenolol toxicity in rabbits showed no significant changes in MAP after giving lipid [49]. While this creates doubt about ILE use in the setting of beta blocker intoxication, the findings may be explained by the fact that atenolol is not nearly as lipophilic as other beta-blockers, such as propranolol. One case report indicated hemodynamic recovery after ILE administration in a patient with both ethanol and atenolol intoxication. However, it cannot be determined whether these improvements were attributed to ILE, atropine, glucagon, or saline [50].

### Other Non-LA Drugs

ILE has been used as a novel treatment approach for other toxicities, including herbacides and pesticides. A recent case of confirmed moxidectin toxicity in a puppy demonstrated vast reduction in recovery time when treated with intravenous lipid over four hours [51]. Dosing of ILE was based on therapeutic recommendations for bupivacaine toxicity, as no such guidelines currently exist for the treatment of non-LA toxicities. The use of ILE in treatment of a patient with refractory hypotension caused by glyphosate-surfactant herbicide (GlySH) has gained considerable attention [52]. Aggressive fluid and vasopressor support did not improve the patient's condition, but the administration of one lipid bolus (100 mL) and subsequent infusion (400 mL) caused a rapid and dramatic return to normal blood pressure. As GlySH is notoriously unresponsive to conventional therapies, the authors suggest that ILE should be considered in such cases of refractory hemodynamic instability.

### Controversies

These animal models and human case reports reveal promising results, but also leave many questions unanswered. Because few animal studies have compared standard resuscitative therapies to treatment with lipid emulsion in non-LA toxicities, future studies need to emphasize the inclusion of ACLS-treated controls.

When patients suffer cardiac or neurologic symptoms from local anesthetic systemic toxicity, the offending agent (a local anesthetic) is known and ILE is a proven resuscitative antidote. However, when patients present in the emergency department with neurologic or cardiac compromise there is the possibility of an unidentified drug overdose. Should the physician administer ILE without the knowledge of what was ingested? What if the suspected toxin is not lipophilic? More studies and better delineation of the mechanisms and limitations of ILE are needed to determine best practices and clinical guidelines for integrating use of ILE with standard resuscitation during non-LA drug overdoses and other potential intoxications.

## Recommendations

ILE should be used in local anesthetic toxicity at the onset of neurological or cardiovascular symptoms. There is no known alternative antidote for the treatment of local anesthetic toxicities resistant to standard ACLS agents. In the setting of other lipophilic drug toxicities causing hemodynamic compromise, when standard resuscitation protocols are unsuccessful, clinicians can consider administration of ILE. While protocols exist for administration of ILE in setting of LAST, no optimal regimen has been established to date for treatment of acute non-LA poisonings.

Weinberg published the first recommendation for the use of ILE in a letter to the editor in 2004 [53]. His 2006 revised version served as the basis for all subsequent recommendations for ILE, including those by the Association of Anaesthetists of Great Britain and Ireland, the American Society of Critical Care Anesthesiologists, the American Society of Anesthesiologists Committee on Critical Care Medicine, and the Resuscitation Council of the UK. Most recently, in spring of 2010, the American Society of Regional Anesthesia (ASRA) published a practice advisory on local anesthetic toxicity, highlighting lipid's role in LAST treatment [54]. These treatment guidelines included the use of ILE as an adjunct to airway management and good CPR, stating "...lipid emulsion therapy can be instrumental in facilitating resuscitation, most probably by acting as a lipid sink that draws down the content of lipid-soluble local anesthetics from within cardiac tissue, thereby improving cardiac conduction, contractility, and coronary perfusion" [54]. A 1.5 mL/kg 20% lipid bolus with subsequent 0.25 mL/kg/minute infusion is the currently recommended protocol. Rebolus and increased infusion may be considered if circulatory stability is not attained, but 10 mL/kg lipid emulsion for 30 minutes is the upper limit recommended for initial dosing. Also, prompt and effective airway management must be implemented to prevent hypoxia and respiratory acidosis, which may potentiate LAST [55].

While use of ILE is now commonplace for treatment of LAST, additional clinical evidence may be needed before ILE can be recommended as a first-line intervention for non-LA overdoses [35]. Local anesthetic induced CNS and CV disturbances are usually witnessed events in the peri-operative environment. These events are discovered quickly and treated expeditiously. In settings such as emergency rooms, the offending drugs must first be determined or estimated before the practitioner can assess whether lipid emulsion would enhance standard resuscitation based on relative measures of lipophilicity.

### Potential Risks of ILE

Recent reports highlight the detrimental effect that standard pharmacologic therapies might have in the setting of lipid emulsion administration [13]. Studies by Weinberg et al in rat models, where bupivacaine overdose caused asystole, showed that lipid was superior to epinephrine [11], vasopressin [12] or the combination of both when hemodynamics were measured at ten minutes. They postulated that severe vasoconstriction and increased lactate levels caused by epinephrine administration may actually exacerbate LAST. A follow up study by Hiller et al confirmed this finding[13]. They used a rat model to demonstrate that adding epinephrine to the lipid infusion at doses above 10 mcg/kg increased lactate concentration, worsened acidosis, and resulted in worse recovery at 15 minutes compared to animals treated with lipid alone. These findings led to the aforementioned 2010 ASRA guidelines that recommend the use of low dose epinephrine, and avoiding vasopressin completely in the setting of LAST.

The side effects of administering large doses of ILE have been evaluated in recent safety studies. In study of the possible pulmonary or neurological complications following high volume 20% lipid infusions in anesthetized rats, results demonstrated both normal tissue histology and a mean LD50 of 67 mL/kg which is one order of magnitude above typical doses [56]. The conclusions support the safety of lipid therapy at amounts recommended by ASRA. It remains unclear whether high doses of lipid could interfere with other administered medications, but no adverse effects have been noted in trials using ILE concomitant with sodium bicarbonate, atropine, or calcium [43,39].

The lack of documented risks of ILE in trials and case reports is encouraging for physicians interested in introducing this therapy to their emergency departments, but cautious interpretation of safety data is advised. The small sample sizes provide insufficient numbers from which to make generalizations about rare or long-term events. Physicians are encouraged to document all cases of ILE utilization (both favorable and unfavorable) at http://www.lipidregistry.org and http://www.lipidrescue.org, as retrospective and prospective data analyses will continue to provide insight into the scope of ILE use. Although randomized controlled trials (RCTs) may not be possible in this field, case reports and animal studies should not suffice as the only source of information. Controlled clinical trials (i.e. treatment given by experimental protocols compared with standard treatment) will be crucial for evaluating the efficacy and potential side effects from lipid emulsion therapy.

## Conclusion

Animal studies and case reports guide our current use of ILE in treatment of both local anesthetic toxicity and non-local anesthetic, highly lipophilic medication toxicity. Controlled clinical trials may be explored as a means of comparing patient outcomes in the future. On the whole, it seems reasonable to assume that a patient in refractory cardiac arrest would suffer little harm if ILE is used as a last attempt in resuscitation. Additional research into the mechanisms of ILE in the effective resuscitation of non-LA drug overdoses will aid the development of clinical guidelines.

Lipid emulsion has been advocated in the resuscitation of local anesthetic toxicity refractory to conventional modes of resuscitation [57]. Based on review of animal studies and the small number of case reports, ILE may be a useful in treatment of non-LA lipophilic medication overdoses as an adjunct to antidotal therapy and ACLS protocols (modified to reduce vasopressor treatment). While not yet considered a generic first-line treatment in the setting of unknown drug overdoses, the use of ILE should be strongly considered, particularly in failed resuscitations. We anticipate that future animal studies and additional case reports will help shape the evolving recommendations for non-LA toxicities.

## Abbreviations

ILE: intravenous lipid emulsion; LAST: local anesthetic systemic toxicity; LA: local anesthetic; EKG: electrocardiogram; RPP: rate pressure product; ACLS: advanced cardiac life support; CNS: central nervous system; ATP: adenosine triphosphate; GCS: Glasgow coma scale; MAP: mean arterial pressure; CCB: calcium channel blocker; CPR: cardiopulmonary resuscitation; CV: cardiovascular; ASRA: American society of regional anesthesia

## Competing interests

The authors declare that they have no competing interests.

## Authors' contributions

LR drafted and revised the majority of the manuscript, including necessary changes. SB edited the manuscript. SO drafted a section of the manuscript. GW supervised, edited, and revised the manuscript for important content. All authors approved the final manuscript.
